# The Emotional Impact of Novel Coronavirus on Healthcare Workers: A Cross-Sectional Study

**DOI:** 10.7759/cureus.26101

**Published:** 2022-06-20

**Authors:** Qurrat Al Ain Atif, Ishfaq Khan, Ahmed M Malik, Adel Hamid

**Affiliations:** 1 Surgery, Darent Valley Hospital, Dartford, GBR; 2 Department of Surgery, Barnsley Hospital, Barnsley, GBR; 3 Renal Transplant, St. George’s Hospital, London, GBR; 4 General Surgery, Aneurin Bevan University Health Board, Newport, GBR

**Keywords:** healthcare workers, emotional impact, pandemic, personal protective equipment, coronavirus

## Abstract

Introduction

Healthcare workers (HCWs) are the foundation of the response to a pandemic. Also termed as frontline workers, not only are they at a health risk but also suffer from emotional and psychological stress.

Objective

The objective of the study was to determine the emotional impact of novel coronavirus on healthcare workers.

Methodology

An online survey was completed by 239 HCWs from five different countries during the peak of the coronavirus disease 2019 (COVID-19) outbreak amidst the lockdown. Their feelings and concerns as well as the safety measures they adopted were identified.

Results

The response rate was 100%. Most of the respondents were 20-40 years old (85.36%) and working as doctors (73.22%); 44.77% were working at middle grade. The majority felt confused (19.67%), whereas others felt stressed/overworked (17.15%), unhappy (16.74%), scared (13.81%), nervous (13.39%), motivated (8.79%), and privileged (5.86%). A few felt pressurized to perform their duty (4.6%), and 69.87% felt that it was their moral obligation to continue their duty, whereas 13.39% felt administrative pressure for the same. Of the respondents, 53.97% feared transferring the disease to their family and friends, while others feared the lack of personal protective equipment (PPE) (13.39%). According to the majority of the respondents (25.94%), support from family and friends had them going through the crisis. The most common safety measure adopted by the HCWs was strict hand hygiene (43.51%). The HCWs (28.87%) felt that adequate and easy access to PPE would have helped them better during the pandemic.

Conclusion

Healthcare institutions are responsible for protecting HCWs or frontline workers during pandemics so they can continue with their duty. From our study, we have concluded that simple protective measures as uninterrupted and easy access to PPE would have helped HCWs deal with their stress and concerns.

## Introduction

Since the severe acute respiratory syndrome (SARS) outbreak in 2003, the 21st century has seen numerous pandemics [1]. Epidemiologically speaking, these infections have no borders to spread because of extensive international travel [2], hence infecting huge numbers all around the globe.

Similarly, the year 2020 has been faced with a new pandemic starting in December 2019 in Wuhan, China, as unusual pneumonia caused by a new coronavirus [3,4]. This is the third outbreak caused by a coronavirus, the first and second being SARS and Middle East respiratory syndrome (MERS), respectively. The novel coronavirus 2019 is officially named SARS-CoV-2 [3]. It was declared a global emergency of international concern by the World Health Organization (WHO) on January 30, 2020 [3].

As of April 28, 2020, the total number of confirmed cases of the disease has been 2,954,222, with 202,597 deaths globally [5]. On the other hand, China alone has had 82,875 confirmed cases and 4,633 deaths as of May 2, 2020 [6].

When the pandemic gained global attention, a sudden decline in personal protective equipment (PPE) supplies [7], startling media reports, a huge influx of patients into hospitals, and a shortage of utilities secondary to bulk buying in lieu of an impending crisis and comparison with previous Coronaviridae outbreaks lead to uncertainty, vulnerability, panic, fear, distress, anger, and feelings of loss of control. Then, social distancing and finally a lockdown were set in place, which made coping with the pandemic even more difficult as people’s financial circumstances changed.

As with any other pandemic, there is a dual effect seen with this virus; not only is there fear and panic in the population but also an increased burden on the healthcare system including healthcare workers (HCWs) [2]. As the experience with SARS showed that HCWs were the most infected with high mortality [1,8-10], fear and uncertainty are markedly present among the HCWs. Other feelings varied from anxiety, to stress, to frustration, to stigmatization [3,11]. Frontline workers were relocated to different departments and were asked to work in different institutions as a part of task force reassignment to deal with the suspected surge, as was previously observed during the SARS outbreak [11].

Frequently changing guidelines on infection control procedures and public health recommendations stirred confusion and anxiety [11].

We wanted to study the emotional impact of novel coronavirus 2019 on HCWs and how they chose to address these concerns.

## Materials and methods

Study design

This prospective, cross-sectional study was conducted using an open online survey filled by HCWs from hospitals caring for COVID-19 patients in the UK, the USA, Pakistan, Libya, and Saudi Arabia. The survey was conducted from April 23, 2020, to May 18, 2020. HCWs from all fields were eligible for participation. The survey was completely anonymous, and responses were kept confidential. The survey was completed by 239 participants. The work has been reported in line with the Strengthening The Reporting Of Cohort Studies in Surgery (STROCSS) criteria [12].

Outcomes

We aimed at assessing the feelings of HCWs during the SARS-CoV-2 pandemic, the reason behind their feelings, how they addressed their concerns, and their suggestions.

Demographic data including age, healthcare category, grade, and department (for physicians and nurses) were recorded and separately used to analyze risk factors.

Statistical analysis

Data analysis was done using the SPSS software version 25.0 (IBM Corp., Armonk, NY, USA).

## Results

Demographic characteristics

**Figure 1:**
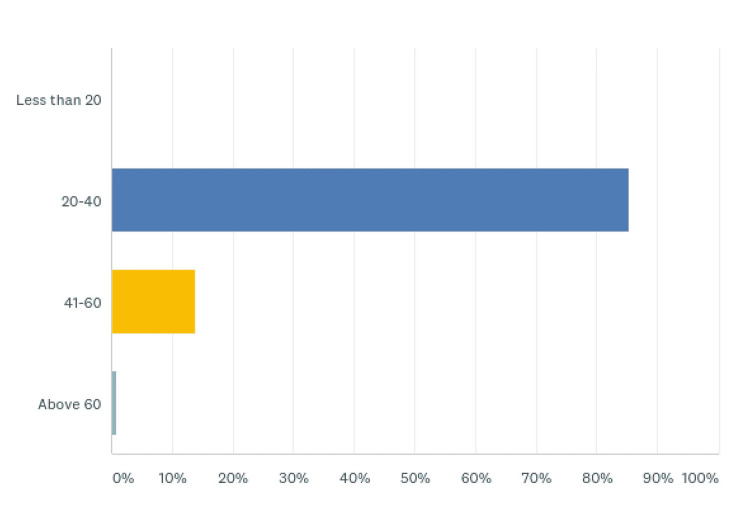
Respondents’ age

Of the 239 participants, the completion rate was 100%. A total of 204 (85.36%) respondents were between 20 and 40 years, 33 (13.81%) between 41 and 60 years, and two (0.84%) more than 60 years (Figure 1, Table 1).

**Table 1:** Respondents’ age

Age	Respondents (%)
Less than 20	0 (0)
20-40	204 (85.36)
40-60	33 (13.81)
Above 60	2 (0.84)

**Figure 2:**
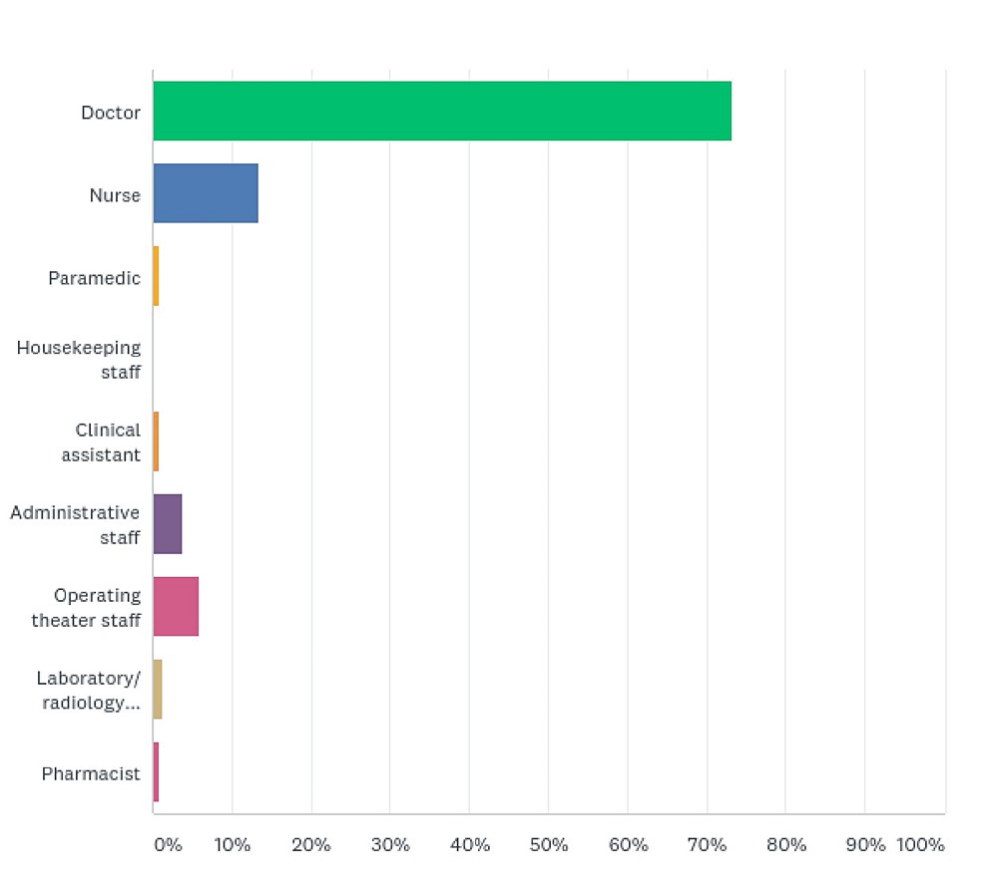
Healthcare category

Of the 239 respondents, 175 (73.22%) were physicians, 32 (13.39%) were nurses, 14 (5.86%) were operating department practitioners (ODPs), nine (3.77%) were administrative staff, three (1.26%) were laboratory/radiology personnel, and two (0.84%) each of paramedics, clinical assistants, and pharmacists (Figure 2, Table 2).

**Table 2:** Healthcare category

Healthcare category	Respondents (%)
Doctor	175 (73.22)
Nurse	32 (13.39)
Paramedic	2 (0.84)
Housekeeping staff	0 (0)
Clinical assistant	2 (0.84)
Administrative staff	9 (3.77)
Operating theater staff	14 (5.86)
Laboratory/radiology personnel	3 (1.26)
Pharmacist	2 (0.84)

**Figure 3:**
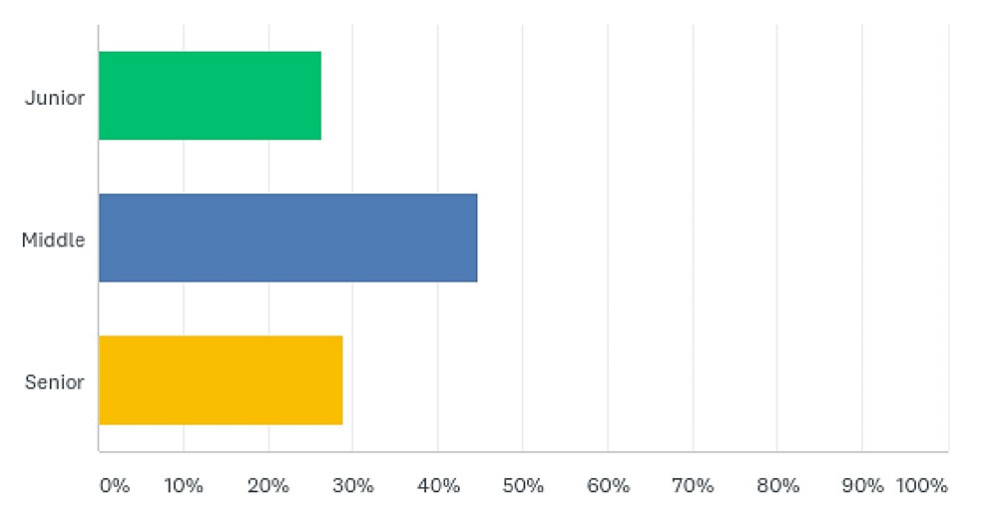
Healthcare category grade

Most of the HCWs belonged to the middle grade (107 (44.77%)), whereas junior and senior grades constituted 63 (26.36%) and 69 (28.87%) of the respondents, respectively (Figure 3, Table 3).

**Table 3:** Healthcare category grade

Grade	Responses (%)
Junior	63 (26.36)
Middle	107 (44.77)
Senior	69 (28.87)

Physicians and nurses were optionally required to record their department. Out of the 175 physicians, 163 answered this question, and the majority of them (55) belonged to general surgery, followed by general practitioners (13), anesthetists (10), and orthopedic surgeons (9). Seven physicians were from medicine; six each from pediatrics, accident and emergency, and otolaryngology; five each from nutrition and radiology; four each from gynecology/obstetrics, dermatology, and neurosurgery; three each from urology, intensive care, and dentistry; two each from pathology, nephrology, and maxillofacial; and one each from infectious diseases, ophthalmology, endocrinology, cardiology, neurology, elderly care, vascular surgery, physical medicine and rehabilitation, and public health.

Outcome measures

**Figure 4:**
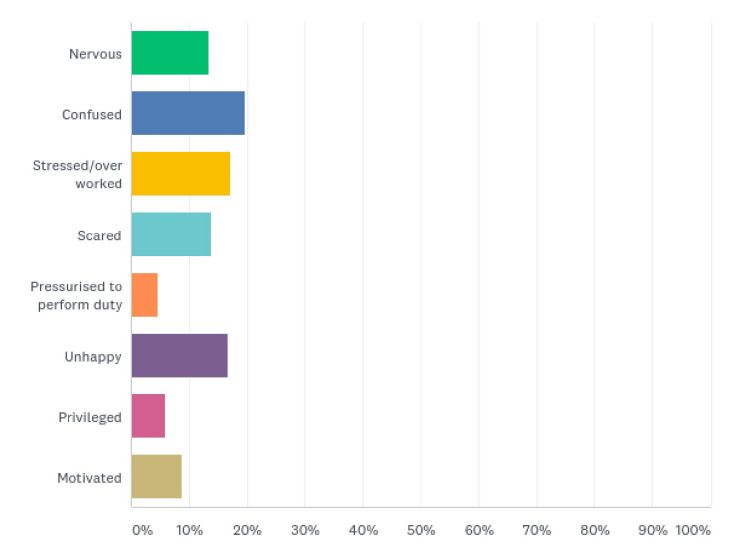
Feelings during the COVID-19 pandemic

The majority of the respondents felt confused (47 (19.67%)) during the pandemic. Forty-one (17.15%) felt stressed/overworked, 40 (16.74%) felt unhappy, and 33 (13.81%) felt scared. Other feelings included feeling nervous (32 (13.39%)), being motivated (21 (8.79%)), being privileged (14 (5.86%)), and feeling pressurized to perform duty (11 (4.60%)) (Figure 4, Table 4).

**Table 4:** Feelings during the COVID-19 pandemic

Feelings	Respondents (%)
Nervous	32 (13.39)
Confused	47 (19.67)
Stressed/overworked	41 (17.15)
Scared	33 (13.81)
Pressurized to perform duty	11 (4.60)
Unhappy	40 (16.74)
Privileged	14 (5.86)
Motivated	21 (8.79)

**Figure 5:**
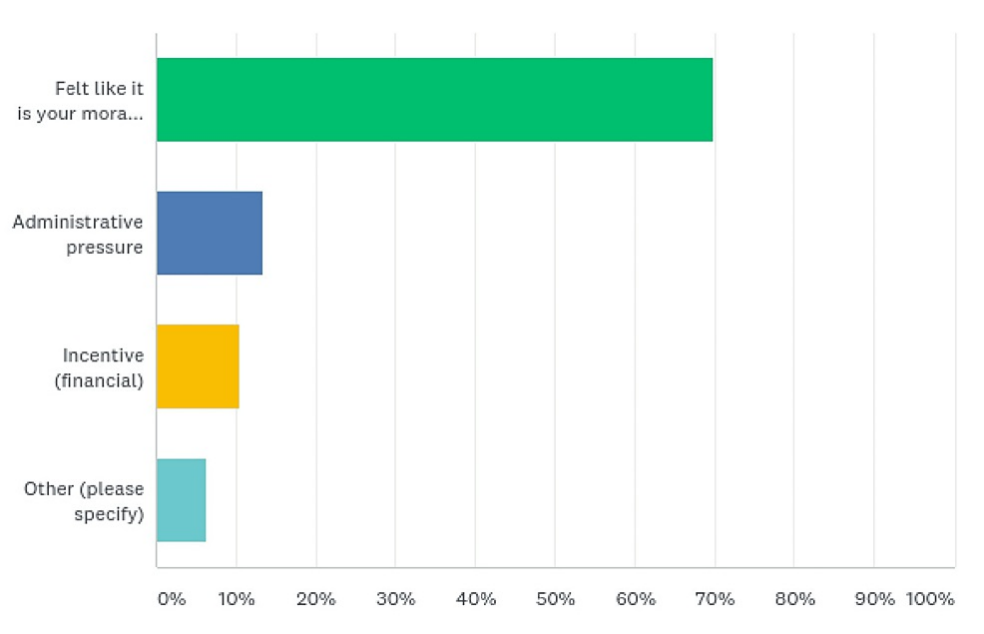
Reason to continue duty

Of the HCWs, 68.87% (167) felt it to be their moral obligation to continue duty, while others felt administrative pressure (32 (13.39%)) for continuation. Twenty-five (10.46%) HCWs chose financial incentives as the main reason to continue working during the pandemic, and 15 (6.28%) had other reasons (Figure 5, Table 5).

**Table 5:** Reason to continue duty

Why did you continue duty?	Responses (%)
Felt like it is your moral duty	167 (69.87)
Administrative pressure	32 (13.39)
Incentive (financial)	25 (10.46)
Other	15 (6.28)

**Figure 6:**
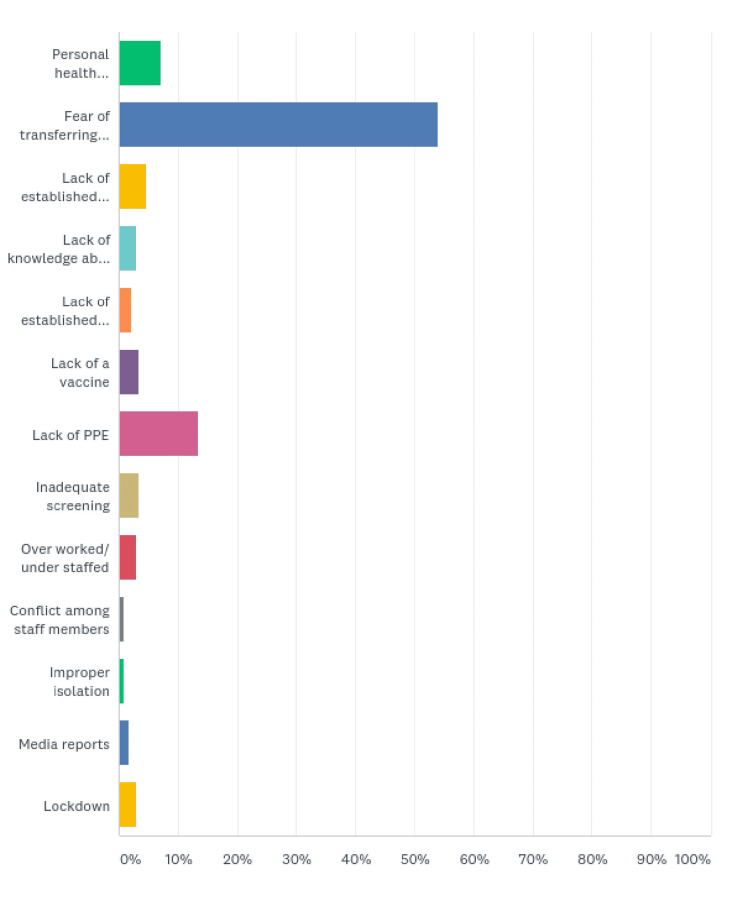
Major concerns

Fear of spreading the disease to their family and friends was prevalent among the HCWs. Overall, 129 (53.97%) respondents chose this as their major concern. Lack of personal protective equipment (PPE) bothered 32 (13.39%) of the respondents. The HCWs were concerned about contracting the disease (17 (7.11%)), lack of established guidelines (11 (4.60%)), lack of a vaccine (8 (3.35%)), inadequate screening (8 (3.35%)), lack of knowledge about the virus or disease (7 (2.93%)), being overworked/understaffed (7 (92.93%)), and lockdown (7 (2.93%)). The lack of established treatment for the disease caused unrest among five (2.09%) of the respondents. Others feared media reports (4 (1.67%)), conflict among staff members (2 (0.84%)), and improper isolation (2 (0.84%)) (Figure 6, Table 6).

**Table 6:** Major concerns

Major concerns	Responses (%)
Personal health (contracting disease)	17 (7.11)
Fear of transferring to family or friends	129 (53.97)
Lack of established guidelines	11 (4.60)
Lack of knowledge about the virus or disease	7 (2.93)
Lack of established treatment	5 (2.09)
Lack of a vaccine	8 (3.35)
Lack of PPE	32 (13.39)
Inadequate screening	8 (3.35)
Overworked/understaffed	7 (2.93)
Conflict among staff members	2 (0.84)
Improper isolation	2 (0.84)
Media reports	4 (1.67)
Lockdown	7 (2.93)

**Figure 7:**
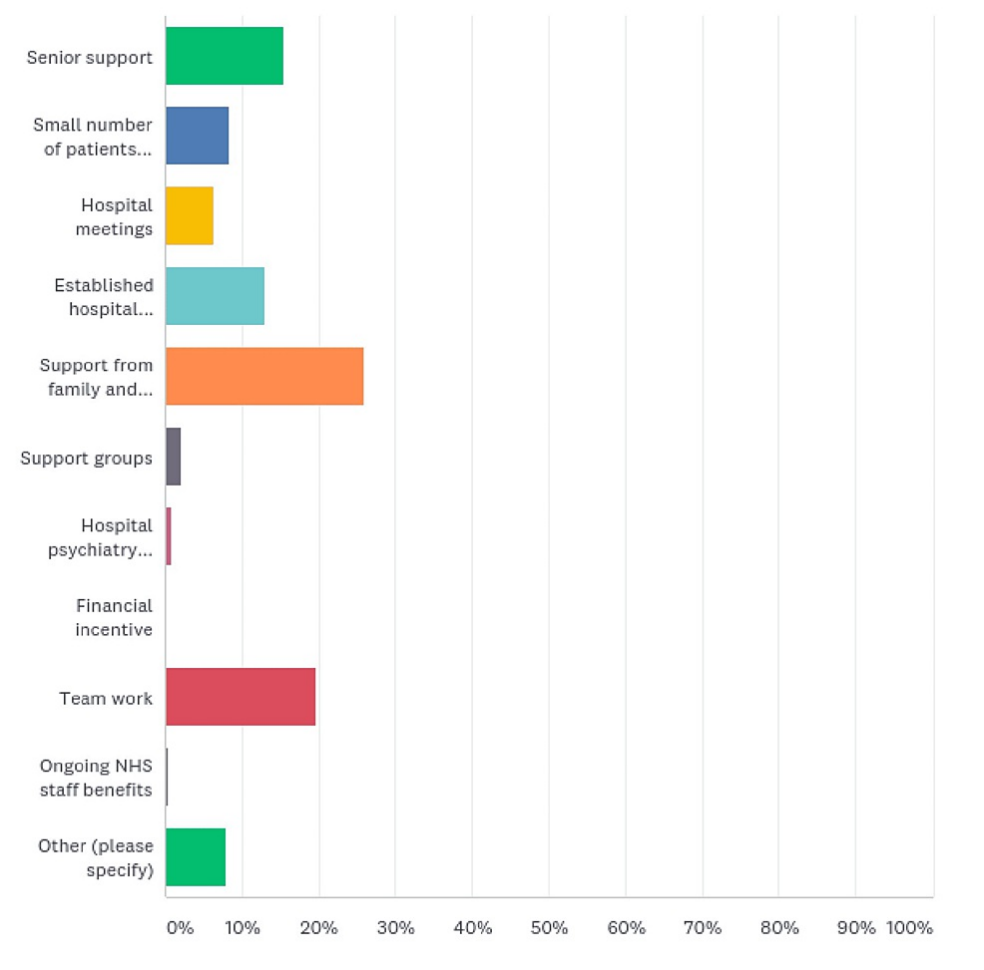
How HCWs addressed their concerns

The responses to how HCWs addressed their concerns included support from family and friends (62 (25.94%)), teamwork (47 (19.67%)), senior support (37 (15.48%)), established hospital guidelines (31 (12.97%)), relatively small number of patients testing positive (20 (8.37%)), hospital meetings (15 (6.28%)), support groups (5 (2.09%)), hospital psychiatry support (2 (0.84%)), and ongoing HCW benefits (1 (0.42%)). A total of 19 respondents had other ways to help them out during the crisis (Figure 7, Table 7).

**Table 7:** How HCWs addressed their concerns

How did you address your concerns?	Responses (%)
Senior support	37 (15.48)
Small numbers of patients tested positive for the disease	20 (8.37)
Hospital meetings	15 (6.28)
Established hospital guidelines	31 (12.97)
Support from family and friends	62 (25.94)
Support groups	5 (2.09)
Hospital psychiatry support	2 (0.84)
Financial incentive	0 (0)
Teamwork	47 (19.67)
Ongoing NHS staff benefits	1 (0.42)
Other	19 (7.95)

**Figure 8:**
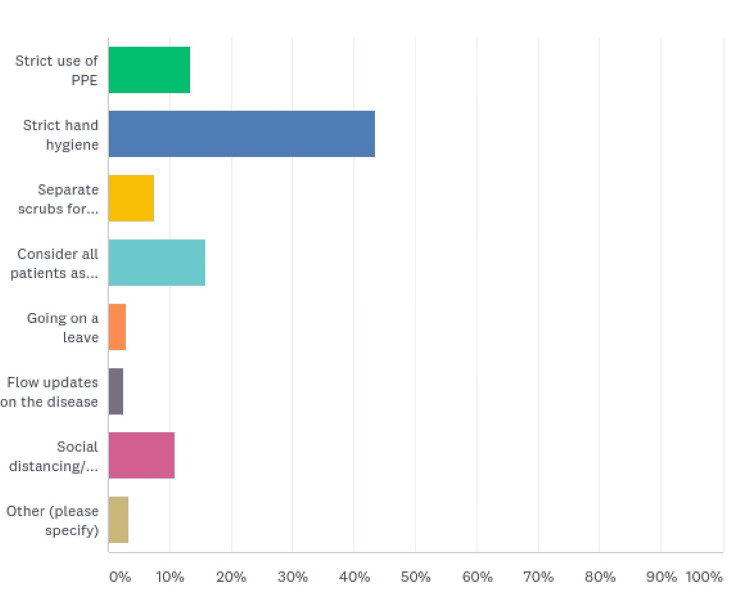
Safety measures adopted by the HCWs

Strict hand hygiene was adopted by 104 (43.51%) HCWs as a safety measure. Thirty-eight (15.90%) considered all patients as carriers, 32 (13.39%) adopted strict PPE use, 26 (10.88%) resorted to self-isolation/social distancing, and 18 (7.53%) had separate scrubs for hospital duty. Seven (2.93%) HCWs went on leave, and six (2.51%) strictly followed updates on the disease. Eight of them adopted other measures (Figure 8, Table 8).

**Table 8:** Safety measures adopted by the HCWs

What safety measures were adopted?	Responses (%)
Strict use of PPE	32 (13.39)
Strict hand hygiene	104 (43.51)
Separate scrubs for hospital	18 (7.53)
Consider all patients as carriers	38 (15.90)
Going on a leave	7 (2.93)
Follow updates on the disease	6 (2.51)
Social distancing/self-isolation	26 (10.88)
Other	8 (3.35)

**Figure 9:**
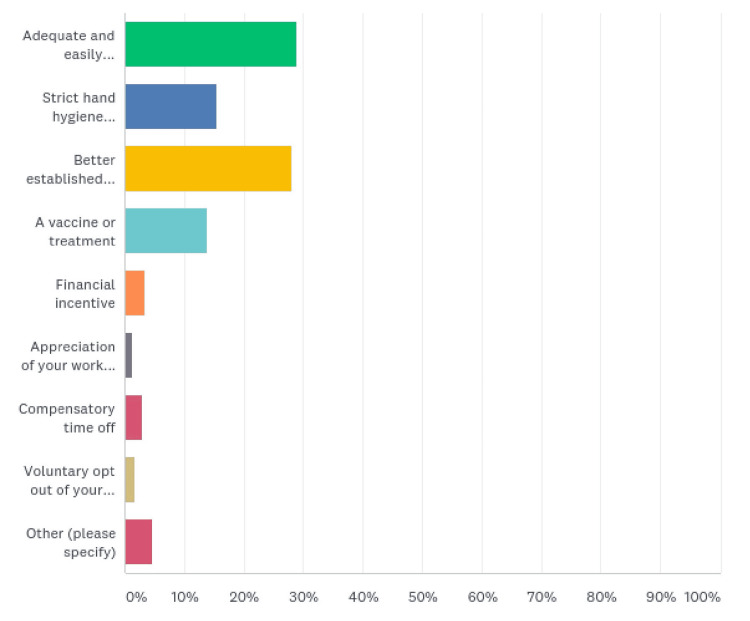
What would have helped to better deal with the situation

When asked what would have helped them better deal with the situation, 69 (28.87%) responded with adequate and easily accessible PPE, and 67 (28.03%) thought better-established guidelines on screening, isolation, and treatment should have been in place. Thirty-seven (15.48%) suggested strict hand hygiene monitoring; for 33 (13.81%), a vaccine or treatment would have been reassuring, while eight ( 3.35%) wished for a financial incentive, seven (2.93%) asked for a compensatory time off, four (1.67%) suggested a voluntary opt-out of duty, and three (1.26%) carved for a little appreciation from authorities. Eleven (4.60%) had other suggestions (Figure 9, Table 9).

**Table 9:** What would have helped to better deal with the situation

What do you think helped?	Responses (%)
Adequate and easily accessible PPE	69 (28.87)
Strict hand hygiene monitoring	37 (15.48)
Better established guidelines on screening, isolation, and treatment	67 (28.03)
A vaccine or treatment	33 (13.81)
Financial incentive	8 ( 3.35)
Appreciation of your work by authorities	3 (1.26)
Compensatory time off	7 (2.93)
Voluntary opt-out of duty or overtime	4 (1.67)
Other	11 (4.60)

## Discussion

Our survey including 239 participants revealed a high prevalence of confusion and stress/being overworked in HCWs involved in the care of COVID-19 patients (19.67% and 17.15%, respectively). This is comparable to studies done during the SARS outbreak [13]. During a pandemic, HCWs are prone to a multitude of feelings [3,11]. Feeling unhappy, scared, nervous, motivated, privileged, and pressurized to perform duty were reported by 16.74%, 13.81%, 13.39%, 8.79%, 5.86%, and 4.60%, respectively. Previous studies during the SARS pandemic reported similar outcomes [3]. The long-term psychological implications of a pandemic have been studied with SARS and need to be kept in mind and assessed during the current pandemic [14,15]. HCWs should be trained in dealing with stress during an infectious outbreak to optimize their response and efficiency.

Doctors formed the majority of the respondents (73.22%), followed by nurses (13.39%). Most of the respondents were between 20 and 40 years of age (85.36%), and 44.77% of them were in the middle grade of their careers.

Another important aspect highlighted by our study was that 69.87% of the HCWs felt motivated to perform their duty during the pandemic despite all the fear, anxiety, and confusion. Because of their direct contact with COVID-19 patients and the fact that this disease has cross communicability [3], 53.97% of the HCWs feared transferring the disease to their family members and friends. Our survey also found out that support from family and friends (25.94%) and teamwork (19.67%) helped HCWs continue to perform their duty despite mounting pressure and fear.

As with any other infectious disease, hand hygiene was opted for by a mere 43.51% of the respondents as the primary safety measure, the numbers not as significant as would have been expected from HCWs. We want to stress the need for further infection control training and strict hand hygiene compliance monitoring for effective infection control and prevention. We would like to suggest that future pandemic response training should include infection control training as an integral part.

Our survey identified that measures as simple as adequate and easily accessible PPE would have made a huge difference in terms of reassuring HCWs as mentioned by 28.87% of the respondents. As observed initially, the sudden shortage of PPE was a rather important factor in causing emotional distress among HCWs as was noted earlier during previous infection outbreaks [7,11]. On the other hand, an almost equal number of respondents (28.03%) thought it would have been better if there were well-established guidelines on screening, isolation, and treatment of COVID-19 patients. Dealing with an unknown pathogen and a rather unfamiliar disease pattern makes it difficult, but diverting resources toward research, as observed during the coronavirus pandemic, was of paramount importance.

## Conclusions

This survey demonstrated the emotional impact of SARS-CoV-2 on HCWs. The mental and emotional well-being of HCWs is of paramount significance if they were to work efficiently. It is the responsibility of healthcare institutions to safeguard their HCWs and provide them with the means to cope with stress and anxiety. Working under stressful conditions during an infectious outbreak would lead to long-term psychological morbidity in HCWs, as previously identified.
